# Generation of High‐Brilliance Polarized γ‐Rays Via Vacuum Dichroism‐Assisted Vacuum Birefringence

**DOI:** 10.1002/advs.202417201

**Published:** 2025-07-10

**Authors:** Chong Lv, Feng Wan, Yousef I. Salamin, Qian Zhao, Mamutjan Ababekri, Ruirui Xu, Jian‐Xing Li

**Affiliations:** ^1^ Department of Nuclear Physics China Institute of Atomic Energy P. O. Box 275(7) Beijing China; ^2^ Ministry of Education Key Laboratory for Nonequilibrium Synthesis and Modulation of Condensed Matter, Shaanxi Province Key Laboratory of Quantum Information and Quantum Optoelectronic Devices, School of Physics, School of Physics Xi'an Jiaotong University Xi'an 710049 China; ^3^ Department of Physics American University of Sharjah Sharjah Sharjah POB 26666 UAE

**Keywords:** high‐brilliance circularly polarized γ‐photon beam, nonlinear compton scattering, unpolarized electron beam

## Abstract

High‐brilliance circularly polarized γ‐photon beams are of great significance for a wide range of applications. However, their generation through nonlinear Compton scattering must require a high‐density longitudinally‐spin‐polarized electron beam and consequently is still a great challenge. Here, a novel method is proposed to generate such γ‐photon beams via the vacuum dichroism (VD)‐assisted vacuum birefringence (VB) effect, only utilizing a well‐established unpolarized electron beam. A linearly polarized laser pulse is splitted into two subpulses with the first one colliding with a dense unpolarized electron beam to generate a linearly polarized γ‐photon beam (via nonlinear Compton scattering), which then further collides with the second subpulse and is transformed into a circularly polarized one via the VB effect. It is found that by manipulating the relative polarization of two subpulses, one can “purify” the polarization of the γ‐photon beam via the VD effect, thereby significantly enhancing the circular polarization of the γ‐photon beam. Due to the VD assistance, the VB effect reaches optimal when the relative polarization is nearly 30°, not the widely used 45° in the common VB detection methods. The numerical results show that one can obtain a circularly polarized γ‐photon beam with average degree of about 30% (43%) for energies above 500 (1000) MeV and brilliance of about 10^24^ (10^23^) photons/(s · mm^2^ · mrad^2^ · 0.1%BW) at 500 (1000) MeV by using a currently feasible laser with a peak intensity of about 10^22^ Wcm^−^2. And, it can be further improved to above 60% (75%) by increasing the laser pulse duration. Moreover, our method is shown to be robust with respect to the laser and electron beam parameters, and can also be used to efficiently confirm the well‐known VB effect itself, which has been predicted a very long time ago but has not been directly observed in experiments yet.

## Graphical abstract

A novel method is proposed for generating such γ‐photon beams via the vacuum‐dichroism‐assisted quantum vacuum birefringence effect, only utilizing well‐established unpolarized electron beams and solving the critical problem of demanding polarized ones. Moreover, the method can serve to confirm the well‐known VB, which is predicted a long time ago but has not been directly observed in experiments yet.

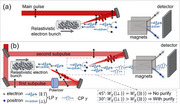

## 1 Introduction

It is conjectured that ultraintense, high‐brilliance, and highly polarized γ‐photons, generated via laser‐based means, will play crucial roles in advancing contemporary research in particle physics,^[^ ^1^ ^]^ nuclear physics,^[^ ^2^ ^]^ astrophysics,^[^ ^3^ ^]^ as well as in many applications in materials science and medicine.^[^ ^4^ ^]^ For example, circularly polarized (CP) γ photons can act as powerful probes for investigating a wide range of fundamental processes that involve spin angular momentum, including parity violation,^[^ ^5^ ^]^ vacuum birefringence (VB), elastic photon‐photon scattering,^[^ ^6^ ^]^ and photoproduction of mesons.^[^ ^7^ ^]^ Currently, high‐energy γ‐photon beams are mainly generated in processes involving synchrotron radiation, bremsstrahlung, and the laser‐Compton effect. Compton scattering of CP light^[^ ^8^, ^9^, ^10^ ^]^ or bremsstrahlung employing longitudinally spin‐polarized (LSP) electrons^[^ ^11^, ^12^ ^]^ can produce CP γ photons. However, the generated beam brilliance is limited due to the small scattering cross‐section and low brilliance of the LSP electron beam produced in conventional accelerators.

Rapid progress in ultraintense laser technology has pushed the peak intensity available to laboratory experiments to the level of *I*_0_ ≃ 10^23^ Wcm^−2^.^[^ ^13^, ^14^, ^15^ ^]^ Thus, high‐brilliance γ‐photon beams can be generated via nonlinear Compton scattering (NCS) of ultraintense laser off dense electron beams produced via laser‐plasma wakefield acceleration (LWFA).^[^ ^16^, ^17^ ^]^ In NCS, the yield of γ photons increases with increasing laser intensity, and the dominant factor of the emission polarization gradually shifts from the polarization of the scattering laser to the electron beam due to the multiphoton absorption effect.^[^ ^18^, ^19^, ^20^ ^]^ Meanwhile, an LSP electron beam is mandatory for generating a highly CP γ‐photon beam via NCS^[^ ^21^, ^22^ ^]^ or bremsstrahlung.^[^ ^23^ ^]^ Also, to attain high‐brilliance γ photons via NCS, the dense LSP electrons can only be generated via LWFA, which is, however, still challenging to maintain the polarization degree of the accelerated electron beam (the polarization will sharply decrease as increasing laser intensity).^[^ ^24^, ^25^ ^]^ At the same time, the circular polarization degree of such γ photons increases linearly with energy, but the yield decreases exponentially; only very high‐energy photons can achieve a high circular polarization degree.^[^ ^22^ ^]^ Thus, it is still a great challenge to generate a high‐brilliance and highly CP γ‐photon beam by a feasible unpolarized electron beam.

The recent advances in laser technologies have also revived theoretical and experimental studies of all‐optical strong field quantum electrodynamics (QED) effects,^[^ ^26^, ^27^ ^]^ such as radiation‐reaction effects,^[^ ^28^, ^29^, ^30^, ^31^ ^]^ NCS,^[^ ^16^, ^17^, ^29^ ^]^ nonlinear Breit‐Wheeler process (NBW),^[^ ^32^ ^]^ and all related polarization effects.^[^ ^21^, ^22^, ^33^, ^34^, ^35^ ^]^ Ultraintense lasers also facilitate exploration of the polarization properties of the quantum vacuum, such as VB^[^ ^36^, ^37^, ^38^ ^]^ and vacuum dichroism (VD).^[^ ^39^, ^40^ ^]^ Conventional detection methods of the VB effect mainly focus on the low‐energy probe photons traversing the magnetically‐polarized vacuum. For instance, Polarization of Vacuum with LASer (PVLAS),^[^ ^41^ ^]^ Observing VAcuum with Laser (OVAL),^[^ ^42^ ^]^ and Biréfringence Magnétique du Vide (BMV)^[^ ^43^ ^]^ use low‐energy photons as probes, and other theoretical proposals also use high‐energy probes.^[^ ^44^ ^]^ However, the detection accuracy is still beyond the attainable signal strength. Petawatt laser‐based VB detection proposals, involving X‐ray free electron lasers (XFEL)^[^ ^45^, ^46^, ^47^, ^48^, ^49^ ^]^ or GeV‐scale γ‐ray probes,^[^ ^40^, ^50^, ^51^, ^52^, ^53^ ^]^ also attract much attention. For instance, VB can be studied by detecting the reduction of linear polarization of the CP γ photons, and vice versa, while VD can be investigated by measuring the change of linear polarization due to *e* ^+^ *e* ^−^ pair production.^[^ ^40^, ^51^, ^54^ ^]^ Recent proposals have also suggested alternative methods for detecting VB, such as Coulomb‐field assistance,^[^ ^55^ ^]^ flying focusing,^[^ ^56^ ^]^ and plasma effects.^[^ ^57^, ^58^ ^]^ Nevertheless, direct observation of the VB effect in experiments with currently available laser systems is still an open question.

**Figure 1:**
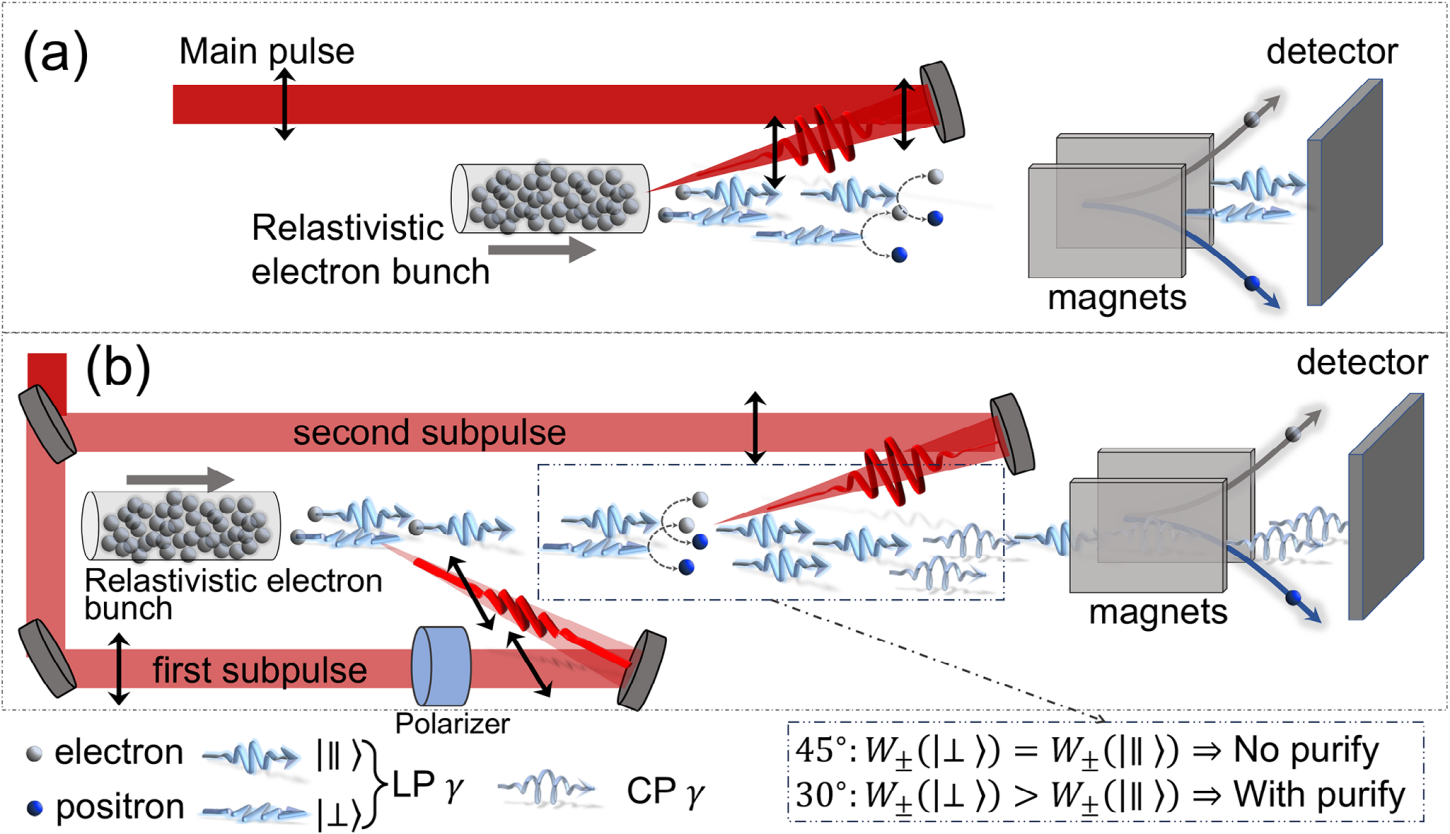
a) Conventional nonlinear Compton scattering and nonlinear Breit‐Wheeler pair production setup. b) Generation of a CP γ‐photon beam via the VD‐assisted VB effect. The main pulse is split into two subpulses. In the first one, the polarization is rotated to θ_<1, 2 >_ = 30° with respect to the second subpulse (main pulse), to conduct the NCS, and the second one is used to collide with the NCS γ‐photons. |∥〉 and |⊥〉 denote photon polarization parallel and perpendicular to the first subpulse, respectively. The pair production rate *W*_±_(|∥, ⊥〉) is asymmetric (symmetric) for θ_<1, 2 >_ = 30° (45°), and thereby with (without) the polarization purification, respectively.

**Figure 2:**
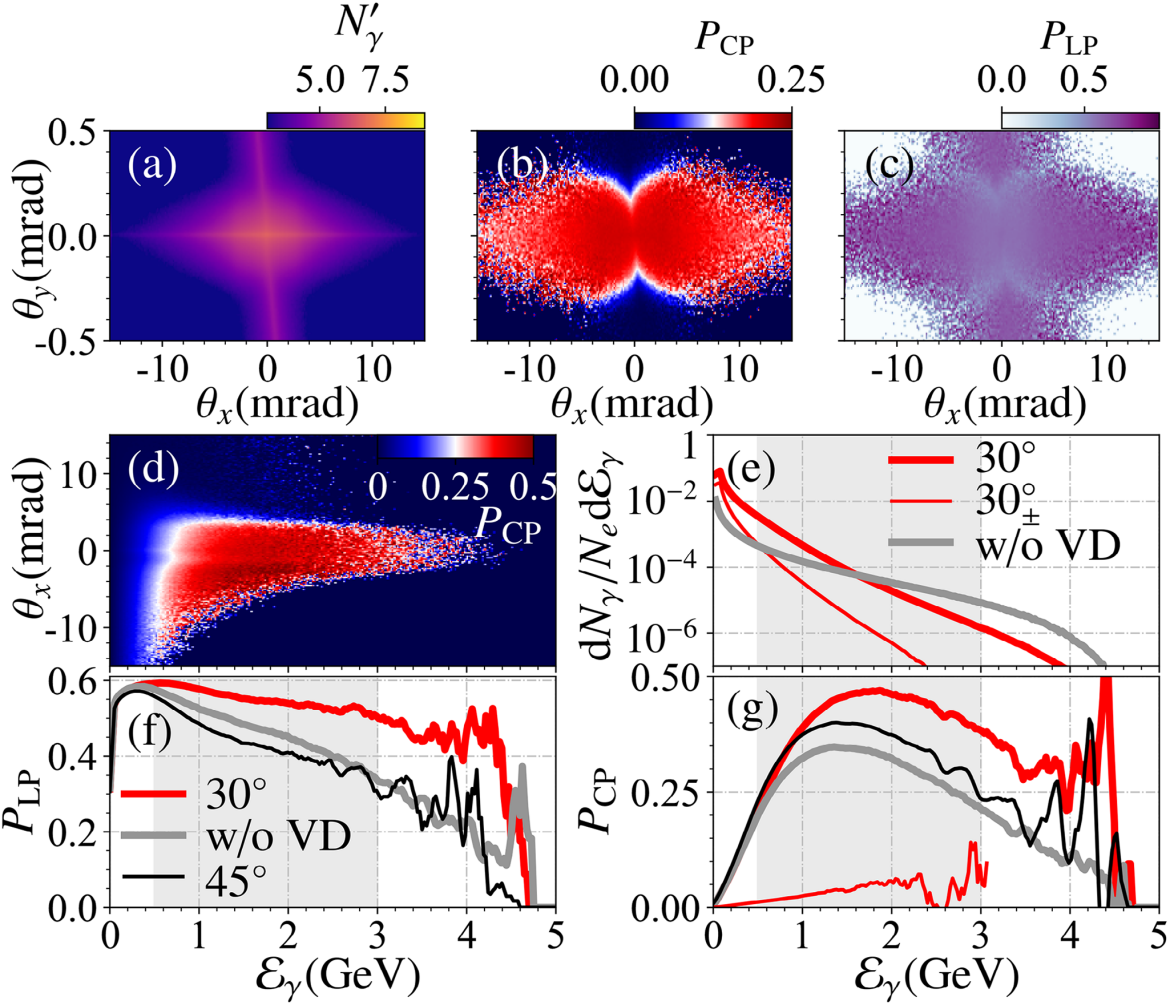
a–c) Photon number density normalized to the electron number and scaled in logarithm $N_{\gamma }^{\prime }\equiv \log_{10}[d$ ^2^(*N*_γ_/*N*_*e*_)/dθ_*x*_dθ_*y*_] (rad^−2^), average circular polarization $P_{\mathrm{CP}}\equiv {\bar{\xi }}_{2}$ and linear polarization $P_{\mathrm{LP}}\equiv {\left({\bar{\xi }}_{1}^{2}+{\bar{\xi }}_{3}^{2}\right)}^{1/2}$ with respect to angles θ_*x*_ and θ_*y*_ in the energy range of 500–3000 MeV (decorated as gray bands in (e)–(g), and $\theta_{x,y}\equiv \arctan\left(\frac{x,y}{z}\right)$). d) *P*_CP_ with respect to the angle θ_*x*_ and energy $E_{\gamma }$. e) Photon energy spectra from primary electrons, *e* ^+^ *e* ^−^ pairs, and without VD (pair production) are denoted as 30°, $30_{\pm }^{\circ }$, and “w/o VD”, respectively. f,g) Energy‐depende nt *P*_LP_ and *P*_CP_ within |θ_*x*_| ≲ 5 mrad and |θ_*y*_| ≲ 0.3 mrad, respectively. Black lines label the results from the case of θ_<1, 2 >_ = 45°, and other lines are the same denotations as those in (e). Due to the low yields of the γ photons, the high‐energy tail presents the poor statistics; see (f) and (g).

**Figure 3:**
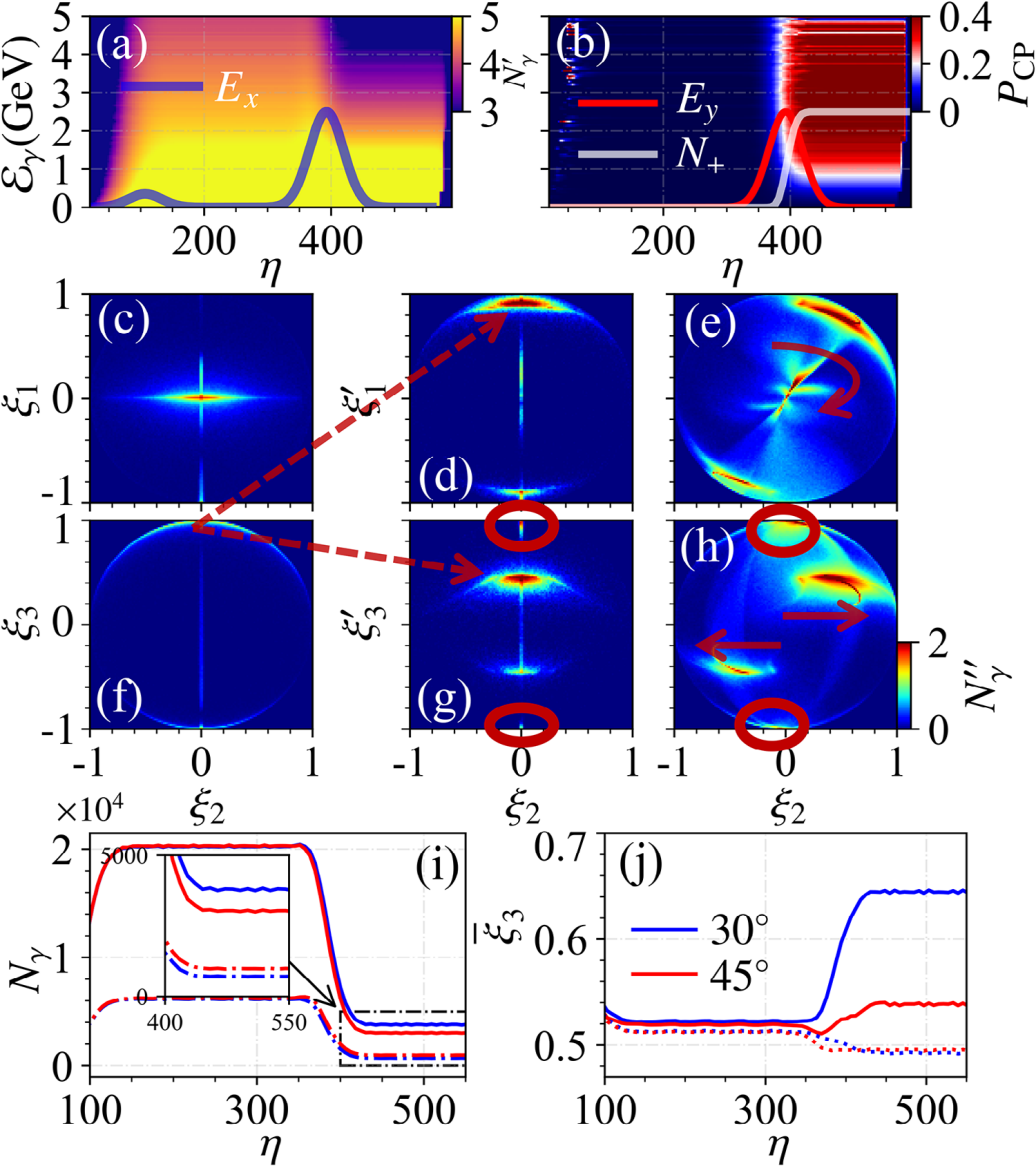
a,b) Photon number density $N_{\gamma }^{\prime }\equiv \log_{10}(d$ ^2^ *N*_γ_/dϕ$dE_{\gamma })$ (a. u.) and *P*_CP_ generated by the primaries. Solid lines indicate the relative temporal profile and η ≡ ω*t* − *kz* is the phase, respectively, of the laser. Photon number distributions *N*″ ≡ log_10_ *N*_γ_(ξ_2_, ξ_1, 3_) (a.u.) with respect to the Stokes parameters of the initial photons in (c) and (f), final photons without VB in (d) and (g), and final photons with VB in (e) and (h), respectively. i) Evolution of photon numbers in a sampled simulation for photons with $E_{\gamma }>1$ GeV, where blue and red lines show results for θ_<1, 2 >_ = 30° and 45°, and solid and dash‐dotted lines represent photons with ξ_3_ > 0 and ξ_3_ < 0, respectively. j) Evolution of ${\bar{\xi }}_{3}$ corresponding to (i), with solid and dotted lines indicating cases of with and without considering the NBW process, respectively. Note that, in (i) and (j), ξ_3_ is taken from the instantaneous frame. Photons in the red circles in (g) and (h) are generated in the second subpulse. Red arrows in (e) and (h) indicate the VB effect.

**Figure 4:**
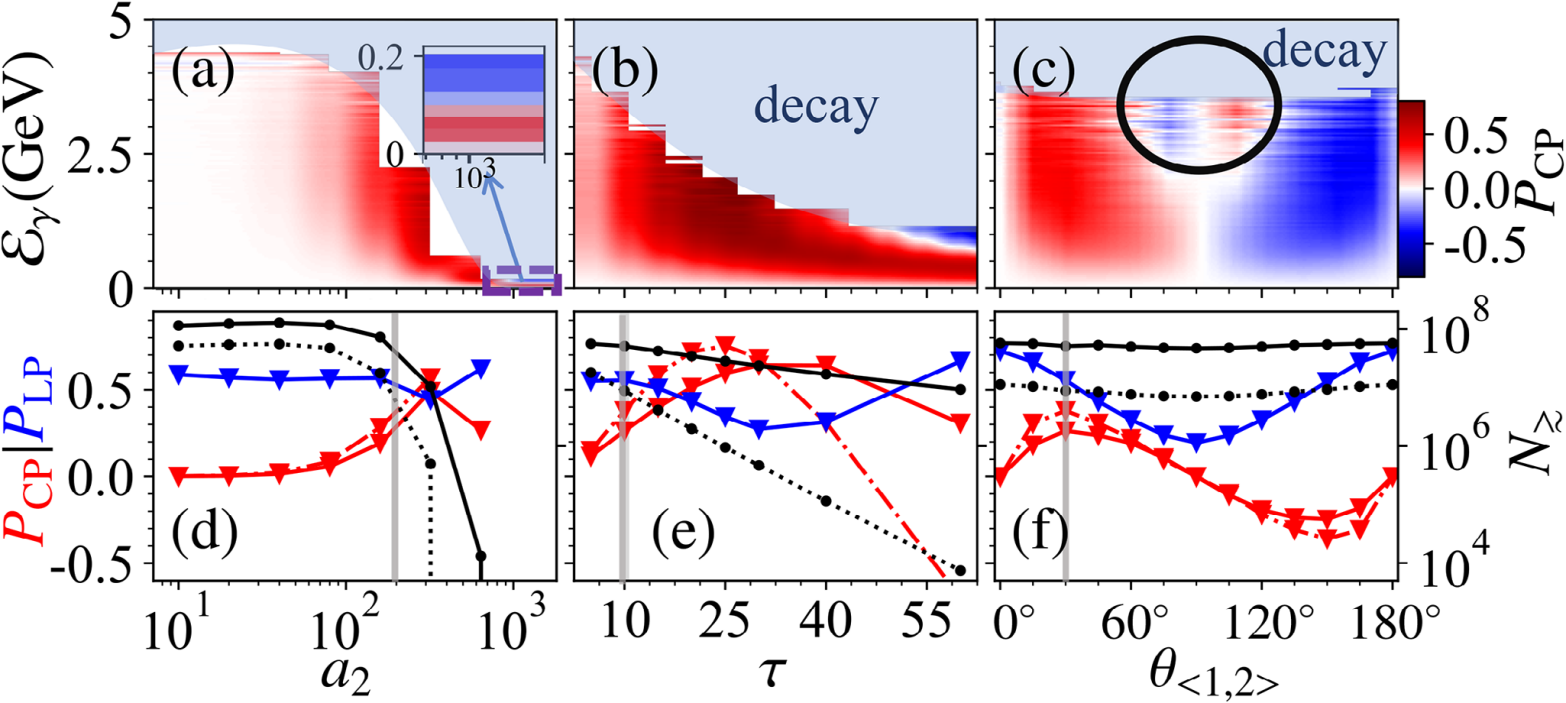
Impact of the laser parameters on the VB effect and polarization degree of the generated γ photon with respect to [(a) and (d)] second subpulse intensity *a*_2_, [(b) and (e)] pulse duration τ, [(c) and (f)] relative polarization angle θ_<1, 2 >_. In the first column, the light‐blue area with “decay” indicates no photons can be detected (due to pair production). The black circle in (c) denotes the anomalous polarization region. In the second column, solid and dash‐dotted lines denote the results of photons with energies $E_{\gamma }≳500$ MeV and 1000 MeV, respectively. All results are collected within an angle of |θ_*x*, *y*_| ⩽ 5 mrad. Red, blue, and black lines indicate *P*_CP_, *P*_LP_, and the total number *N*_≳_. The vertical gray lines indicate parameters used in Figures 2 and 3. All other parameters are the same as those in Figure 2.

In this work, we put forward a novel method to generate high‐brilliance and highly polarized γ‐photon beams via the VD‐assisted VB effect, only utilizing a well‐established unpolarized electron beam; see **Figure** **1** for a schematic. In the conventional setup in Figure 1a, an intense linearly polarized (LP) laser pulse colliding with a relativistic electron beam only generates brilliant LP γ photons via NCS. By contrast, in our proposed setup in Figure 1b, an LP laser pulse is split into two subpulses: the first one is used to perform NCS and generate brilliant LP γ photons; the second one is used to partially convert the LP γ photons into the CP ones via the VB effect (to ensure sufficient photon production in the first subpulse and significant VB effect in the second subpulse, the second subpulse is chosen to be relatively stronger than the first one). Meanwhile, as shown in the dash‐dotted box in Figure 1b, by fine‐tuning the polarization angle θ_<1, 2 >_ between two subpulses, we find that NBW‐induced VD effect can enhance the polarization of the γ‐photon beam by changing the relative numbers of photons in different modes |∥〉 and |⊥〉, i.e., indirectly “purifying” the beam polarization. The circular polarization degree can reach about 30%(43%) for energies above 500(1000) MeV and brilliance of about 10^24^(10^23^) photons/(s · mm^2^ · mrad^2^ · 0.1%BW) at 500 (1000) MeV; see details in **Figure** **2**. And, it can be further improved to 75% by increasing the laser pulse duration. Note that, the optimal θ_<1, 2 >_ is about 30°, not the 45° used in the common VB detection methods;^[^ ^40^, ^50^, ^51^ ^]^ see detailed explanations in **Figure** **3**. Moreover, the circular polarization degree with respect to θ_<1, 2 >_ can serve as a reliable indicator of the VB effect. In addition, our method is shown to be robust with respect to the laser and electron beam parameters; see **Figure** **4** in Section of the Experimental feasibility.

## 2 Methods and Results

To simulate the interactions in Figure 1b self‐consistently, the spin‐resolved NCS and NBW are investigated by our Monte‐Carlo methods,^[^ ^22^, ^59^, ^60^, ^61^ ^]^ incorporating with the VB effect by evolution of the Stokes parameters.^[^ ^40^, ^58^, ^61^ ^]^ Importance of the NCS, NBW, and VB effects is signified by the electron/photon nonlinear quantum parameter $\chi_{e,\gamma }=e\hbar /\left(m_{e}^{2}c^{3}\right)\sqrt{{\left(F^{\mu \nu }p_{\mu }\right)}^{2}}≃2E_{e,\gamma }/\left(m_{e}c^{2}\right)a_{0}\left(\hbar \omega_{r}/\left(m_{e}c^{2}\right)\right)$, where ℏ, −*e*, *m*_*e*_, and *c* are the reduced Planck's constant, charge and mass of the electron, and the light speed in vacuum, respectively, E and *p*_μ_ are energy and four‐momentum vector of the particle. *F* ^μν^, *a*_0_ ≡ *eE*/(*m*_*e*_ *c*ω_*r*_), and *E* are the field tensor, peak intensity, and electric field strength of the laser, respectively, and ω_*r*_ the reference frequency used for normalization. To describe the VB effect in a wide regime of χ_γ_, we use the accurate refractive indices described below.

When an electron (positron) interacts with the laser field and generates a γ photon via NCS, a set of Stokes parameters $\xi =(\xi_{1},\xi_{2},\xi_{3})$ with respect to polarization basis ${\hat{e}}_{1}$ and ${\hat{e}}_{2}$ are assigned to the photon.^[^ ^62^, ^63^ ^]^ Here, ${\hat{e}}_{1}∥E_{\mathrm{red}.,⊥}$, ${\hat{e}}_{2}∥\hat{k}\times {\hat{e}}_{1}$, $\hat{k}$ is the direction of the photon momentum and $E_{\mathrm{red}}.\equiv \left(E+\hat{k}\times B\right)$ is the reduced electric field.^[^ ^64^ ^]^ ξ_3_ indicates the linear polarization parallel to ${\hat{e}}_{1}$, ξ_1_ linear polarization parallel to the direction with π/4 relative to ${\hat{e}}_{1}$, and ξ_2_ the circular polarization.^[^ ^65^ ^]^ In the subsequent evolution and pair production, the eigenbasis should be obtained with the local field. When the eigenbasis rotates by δψ, the Stokes parameters transform via a rotation matrix $\left(\begin{array}{c} \xi_{1}^{\prime }\\ \xi_{3}^{\prime } \end{array}\right)=\left(\begin{array}{cc} \cos2\delta \psi & -\sin2\delta \psi \\ \sin2\delta \psi & \cos2\delta \psi \end{array}\right)\left(\begin{array}{c} \xi_{1}\\ \xi_{3} \end{array}\right)$,^[^ ^40^, ^44^ ^]^ where primed and unprimed Stokes parameters are evaluated in the updated and original frames, respectively. Note that primed ones will denote Stokes parameters in the second subpulse in the latter. Before the photon decays into an *e* ^+^ *e* ^−^ pair, its Stokes parameters evolve due to the VB effect. Within a single time step, the evolution can be represented by a phase rotation $\left(\begin{array}{c} \xi_{1}^{f}\\ \xi_{2}^{f} \end{array}\right)=\left(\begin{array}{cc} \cos\delta \phi & -\sin\delta \phi \\ \sin\delta \phi & \cos\delta \phi \end{array}\right)\left(\begin{array}{c} \xi_{1}^{i}\\ \xi_{2}^{i} \end{array}\right)$,^[^ ^44^ ^]^ where *i* and *f* indicate the initial and final Stokes parameters of the γ photon. Also, $\delta \phi =\int_{0}^{L}dl\left(2\pi /\lambda_{\gamma }\right)\Delta n\left(\omega_{\gamma }\right)$ is the phase delay between two eigenstates for a photon (wavelength λ_γ_ and frequency ω_γ_) traversing a polarized vacuum of length *L*, and Δ*n* = *n*_⊥_ − *n*_∥_, with ∥ and ⊥ denoting the polarization modes parallel to ${\hat{e}}_{1}$ and ${\hat{e}}_{2}$, respectively. *n*_∥, ⊥_ follow from the real and imaginary parts Re(n)=1−454D∫01dυf(υ)πx4/3Gi′(x2/3) and Im(n)=454D∫01dυf(υ)x23K2/323x of the refractive index *n*.^[^ ^40^, ^58^, ^66^ ^]^ Here, $D=\frac{\alpha }{90\pi }{\left(\frac{e|E_{\mathrm{red},⊥}|}{m_{e}^{2}}\right)}^{2}\equiv \frac{\alpha }{90\pi }\frac{\chi_{\gamma }^{2}}{\omega^{2}/m_{e}^{2}}$, $f\left(\upsilon \right)=\left(1-\upsilon^{2}\right)\left\{\begin{array}{c} \frac{1}{2}\left(1+\frac{1}{3}\upsilon^{2}\right)\\ 1-\frac{1}{3}\upsilon^{2} \end{array}\right\}$, with Gi′(*x*) being the derivative of the Scorer function, K_ν_(*x*) the modified Bessel function of the second kind, and *x* = 4/(1 − υ^2^)χ_γ_. The first and second rows of *f*(υ) denote the polarization states parallel to ${\hat{e}}_{1}$ and ${\hat{e}}_{2}$, respectively, i.e., labeled with ∥ and ⊥. Re(*n*) accounts for the photon propagation, and Im(*n*) accounts for the *e* ^+^ *e* ^−^ pair production, which is resolved by the NBW process.^[^ ^54^, ^64^, ^66^ ^]^ In each time step for γ‐photon propagation, the Stokes parameters will first be mapped to the instantaneous frame of ${\hat{e}}_{1}$, ${\hat{e}}_{2}$, and the NBW is checked. If no pairs are created, the polarization may change due to the selection effect,^[^ ^67^ ^]^ and then the photon will continue to propagate including the VB‐induced phase delay, i.e., rotating the Stokes parameters. Since the electron bunch is ultrarelativistic and the laser field strength (*a*_0_ ≃ 30–200) significantly exceeds the Coulomb field of electrons and positrons (see the PIC simulation in SM^[^ ^68^ ^]^), the process is simulated using a single‐particle code that incorporates cascade generation. Meanwhile, due to the field strength is ultrarelativistic, linear QED processes are ignored.^[^ ^18^, ^69^, ^70^ ^]^

As an example, the total intensity of the laser is *I*_0_ ≃ 5.48 × 10^22^ W·cm^−^2 (corresponding to *a*_0_ ≃ 200), pulse duration τ = 10*T*_0_ (*T*_0_ = λ/*c*), focal radius *w* = 3 µm and wavelength λ = 1 µm. The first subpulse is *a*_1_ = 30 and the second one with $a_{2}=\sqrt{a_{0}^{2}-a_{1}^{2}}≃197.7$. The main pulse is split into two subpulses by using a beamsplitter before the compressor, and then two subpulses can be injected into two independent compressors.^[^ ^31^, ^71^ ^]^ As two subpulses share the same oscillator and amplifier, it can avoid the problem of jitter in the time synchronization.^[^ ^71^ ^]^ Moreover, the synchronization of two subpulses can be further optimized by using the optical synchronization techniques.^[^ ^72^ ^]^ Two subpulses are arranged on the two sides of the electron beam. And the collision distance between the γ photons generated in the first subpulse and second subpulse could be flexibly adjusted. Besides, we have also estimated the number of photons could be witnessed by the second subpulse with respect to the distance between the two subpulse focal position; see results in SM.^[^ ^68^ ^]^ The polarization of the first subpulse is then rotated to 30° with respect to the original polarization (the second one). We use a feasible electron beam with a peak energy of 5 GeV, energy spread of 3%, angular spread of 0.1 mrad, longitudinal size of 5 µm, transverse radius of 0.4 µm, and number of 6.25 × 10^7^ (total charge of 10 pC).^[^ ^73^ ^]^ In the absence of the dense LSP electron beams from LWFA in experiments, here we employ the well‐generated unpolarized electron beams.

In all simulations, the first subpulse is assumed to be polarized along the *x*‐axis. The maximum photon yield per electron can reach 10^8^/rad^2^; see Figure 2(a). The peak degrees of circular and linear polarization can reach about 45% and 73%, respectively; see Figure 2b–d. See the full energy range version in Supporting Information.^[^ ^68^ ^]^ In certain applications (e.g., photoproduction of π^0^ pairs from nucleons^[^ ^74^ ^]^ and deuteron photodisintegration^[^ ^75^ ^]^), one can obtain about 8 × 10^6^ (4 × 10^7^) γ photons per shot with $E_{\gamma }≳1000$ MeV (500 MeV) and a circular polarization degree of approximately 43% (30%) [Figures 2e,g and 4]. The circular and linear polarization of the photons is unlikely to be separated via the angle filtering as the circular one originates from the linear one via the VB effect; see Figure 2b,c. Fortunately, when the laser pulse duration increases properly, the circular polarization can be enhanced, and even higher than the linear one; see Figure 4e. Moreover, when θ_<1, 2 >_ ≃ *n*π (*n* is an integer), the beam is highly LP with a degree of about 80%, which is much higher than directly obtained via the NCS;^[^ ^22^ ^]^ see Figure 4f. Note that the VD effect will consume high‐energy photons, which decay into *e* ^+^ *e* ^−^ pairs, and these pairs can further emit low‐energy photons, while both the circular and linear polarization can be enhanced; see Figure 2e,f,g. In addition, the circular polarization is mainly contributed by photons generated by primary electrons in the first subpulse; see Figure 2g. Secondary photons from NBW *e* ^+^ *e* ^−^ pairs generated in the second subpulse, can barely be CP (with a degree of about 1% − 2%).

As shown schematically in Figure 1b, the generation of high‐energy CP γ photons is accomplished in two successive stages: NCS in the first subpulse, followed by VB (accompanied by NBW and NCS cascade) in the second subpulse. In the first stage, only primary electrons emit photons, and the NBW pair production is negligible due to the low intensity of the first subpulse; see the evolution of photon spectra in Figure 3a and normalized positron number *N*_+_ in Figure 3b. Since radiation loss by an electron is proportional to the laser energy via $\delta \gamma_{e}\propto a^{2}\gamma_{e}^{2}\omega^{2}\tau_{L}$,^[^ ^28^ ^]^ with a stronger scattering laser more photons are emitted by primary electrons in the second stage; see Figure 3a, but with much lower energies than in the first stage. In the second stage, high‐energy γ photons can create *e* ^+^ *e* ^−^ pairs via the NBW process; see Figure 3b. These pairs, in turn, can emit abundant photons which is negligible for the final photon energy spectrum and polarization in the energy range of $E_{\gamma }>500$ MeV; see Figure 2e,g. Besides, further cascade is suppressed due to the decrease of χ.

## 3 Discussions

### 3.1 Physical Analysis of the VB Signal

All these photons are initially LP along the external field (either in the first or second subpulse) with an average Stokes parameter of ξ_3_ ≃ 0.6 ($E_{\gamma }/E_{e}≃0$) and then decreasing almost linearly to 0 for $E_{\gamma }/E_{e}≃1$.^[^ ^22^ ^]^ In the whole energy range, one has ${\bar{\xi }}_{1}≃{\bar{\xi }}_{2}≃0$; see Figure 3c. For photons emitted in the first stage, there is an angle δψ = θ_<1, 2 >_ between the instantaneous eigenbasis and the polarization of the second subpulse. Subsequently, due to the change of the instantaneous eigenbasis, their Stokes parameters change as well when they enter the second subpulse; see Figure 3d,g, where photons are redistributed from ξ_3_ ≃ ±1 to $\xi_{1}^{\prime }≃\pm \sin2\theta_{<1,2>}≃\pm \sqrt{3}/2$ and $\xi_{3}^{\prime }≃\pm \cos2\theta_{<1,2>}≃\pm 1/2$; see the arrow indication from Figure 3f to Figure 3d,g for the case of ξ_3_ ≃ +1. In principle, to obtain high circular polarization via the VB effect, $\xi_{1}^{\prime }$ should acquire a maximum with θ_<1, 2 >_ = ±π/4 (45°), and $\xi_{3}^{\prime }≃0$. When these photons propagate in the second subpulse, some of them decay into *e* ^+^ *e* ^−^ pairs, and others experience the VB effect, i.e., $\xi_{1}^{\prime }$ transfers to ξ_2_; see the rotation and translation in Figure 3e,h. However, for photons emitted in the second subpulse, the polarization basis at the instant of creation, and during propagation or pair‐production, is the same (${\hat{e}}_{1}∥E_{\mathrm{red}.,⊥}$ and δψ = 0). Thus, for these photons, ${\bar{\xi^{\prime }}}_{1}≃0$, and the VB effect is negligible (VB only transfers polarization between $\xi_{1}^{\prime }$ and ξ_2_); see these photons in the red circles of Figure 3g,h. Besides, since the energy of photons generated in the second subpulse is relatively smaller than that of those generated in the first subpulse, they affect the final circular polarization degree negligibly; see Figure 3a. To ensure more photons can scatter with the second subpulse, the scattering point between two subpulse should be within several centimeters, and therefore, deplection of the primary electrons is limited; see photon distribution before scatters with the second subpulse in ref. [68]. Fortunately, the deflection of the primary electrons before scattering with the second subpulse does not significantly affect the final circular polarization; see Figure S4 (Supporting Information) in ref. [68].

An interesting finding that sounds counterintuitive is the optimal angle between the two subpulses is not 45° which is usually used in the common VB detection methods.^[^ ^40^, ^50^, ^51^ ^]^ After embedding the QED cascade of NCS and NBW, the simulations show that the case of θ_<1, 2 >_ = 30° can yield a much higher circular polarization degree than that in the case of 45°; see Figure 2g. This contradiction arises when the polarization‐dependent NBW acts as a purifier of the photon polarization, i.e., the VD effect. The circular (linear) polarization degree of NCS photons could increase by over 30% (20%); see Figure 2f,g. This increment in the circular polarization degree is not an expected result in the common VB or VD detection proposals, where an exponential decay $e^{-(\lambda_{1}+\lambda_{2})}$ is presented before all Stokes parameters due to the VD effect (λ_1, 2_ > 0 are related to the pair production rate).^[^ ^40^ ^]^ As indicated in Figure 1b, this VD‐assisted purifying mechanism stems from the NBW polarization‐dependent pair‐production rate, which has the form of $W_{\pm }\propto W_{0}+\xi_{3}^{\prime }W_{3}$, with *W*_0_ only depending on the photon energy and *W*_3_ being the polarization‐dependent term.^[^ ^63^ ^]^ For photons generated by NCS, the average polarization is $\bar{\xi_{3}}≃60\%$ for the low energy range and ≃ 0 for the high energy range. This means that for low‐energy photons, about 20% and 80% of photons acquire ξ_3_ = −1 and 1, and for high‐energy photons, about 50% and 50%, respectively. When these photons enter the second subpulse, the Stokes parameters get rotated into the new frame with $\xi_{3}^{\prime }=\cos2\theta_{<1,2>}\xi_{3}$ and $\xi_{1}^{\prime }=\sin2\theta_{<1,2>}\xi_{3}$. Therefore, for θ_<1, 2 >_ = 45° ($\bar{\xi_{3}^{\prime }}≃0$), the pair‐production rate is symmetric for $\xi_{1}^{\prime }=\pm \sin2\theta_{<1,2>}≃\pm 1$. However, for θ_<1, 2 >_ ≠ 45° ($\bar{\xi_{3}^{\prime }}\neq 0$), it is no longer symmetric for $\xi_{1}^{\prime }≃\pm \sin2\theta_{<1,2>}$; see Figure 3i. More photons with ξ_3_ > 0 and fewer ones with ξ_3_ < 0 are left for the 30° than 45°. This asymmetric pair‐production rate results in the increase of $\bar{\xi_{1}^{\prime }}$ and therefore can produce higher $\bar{\xi_{2}}$; see the final $\bar{\xi_{3}}$ ($≃\bar{\xi_{1}^{\prime }}/\sin2\theta_{<1,2>}$) in Figure 3j. In principle, this angle can be optimized via the *n*‐step pair‐production rate $\max\left(\bar{\xi_{1}^{\prime }}\right)=\max\left(\xi_{1}^{\prime }\frac{N_{+}-N_{-}}{N_{+}+N_{-}}\right)=\max\left(\sin2\theta_{<1,2>}\frac{N_{+}-N_{-}}{N_{+}+N_{-}}\right)$, where *N*_±_ and $N_{\pm }\equiv N_{\pm }{\left[1-\left(W_{0}-\xi_{3,\pm }^{\prime }W_{3}\right)\right]}^{n}=N_{\pm }\left[1-\left(W_{0}\mp \cos2\theta_{<1,2>}W_{3}\right]\right)]$ denote the total and nondecay photon numbers with ξ_3, ±_ = ±1, respectively. With current laser and electron parameters, owing to the VD enhancement to the polarization of NCS photons, we obtain the optimal θ_<1, 2 >_ ≃ 30°, not the 45° used in the common VB detection methods.^[^ ^40^, ^50^, ^51^ ^]^

### 3.2 Experimental feasibility

To demonstrate the experimental feasibility of our method, we focus on the dimensionless intensities *a*_1, 2_ of the two subpulses, pulse duration τ, and their relative polarization angle θ_<1, 2 >_. For a specific laser facility, the total intensity is fixed, e.g., *a*_0_ ≃ 200. By varying the relative intensity of both subpulses, the yield number is quite stable, but the circular polarization degree decreases for *a*_1_ ≳ 100; see in ref. [68]. For the total energy being fixed, by varying the pulse duration τ, the yield number and the circular polarization degree are quite stable within a pulse duration range of about 5‐30*T*_0_ for energies $E_{\gamma }≳500$ MeV; see in ref. [68]. For a fixed *a*_1_ and a varied *a*_2_ over a wide range, the circular polarization degree continuously increases until *a*_2_ ≃ 400 and more photons are converted into pairs. Increasing *a*_2_ further induces the VB phase retardation δϕ ≳ π for high‐energy photons, and one has $\xi_{2}≃\xi_{1}^{\prime }\sin\delta \phi <0$; see Figure 4a. This pattern also applies to the pulse duration τ. In the second subpulse, the linear polarization can be transferred to the circular one, i.e., $\xi_{1}^{\prime }$ is transferred to ξ_2_ due to the VB effect via $\xi_{2}\propto \xi_{1}^{\prime }\sin\delta \phi$, with $\delta \phi \propto \frac{\int_{0}^{L}2\pi \Delta n\left(\omega \right)}{\lambda_{\gamma }}dl\propto \frac{\Delta n(\omega_{\gamma })\tau }{\lambda_{\gamma }}$, and *L*∝*c*τ in the external field. For δϕ ≪ 1, one has $\xi_{2}\propto \xi_{1}^{\prime }\delta \phi \propto \xi_{1}^{\prime }\tau$, i.e., the circular polarization is proportional to the pulse duration of the second subpulse; see cases with τ ≲ 20*T*_0_ in Figure 4e. While for much longer pulse durations, on the one hand, $\xi_{2}\propto \xi_{1}^{\prime }\sin\delta \phi$ is periodic with respect to the phase retardation δϕ. On the other hand, more photons will decay into pairs. These two effects will both decrease the circular polarization degree; see cases of τ ≳ 30*T*_0_ in Figure 4e. And high‐energy photons acquire negative ξ_2_ for larger τ. Meanwhile, as the laser pulse is focused, due to the diffraction, the *e* ^+^ *e* ^−^ pair production and photon emission in the second subpulse is mitigated comparing with plane‐wave subpulse; see in ref. [68]. The optimal pulse duration of the currently available laser facility is in the range of 10*T*_0_ − 30*T*_0_. In the case of τ = 25*T*_0_, the circular polarization degree can reach 60% and 75% for photon energy over 500 and 1000 MeV, respectively. Relative polarization angle θ_<1, 2 >_ of the two subpulses is another key parameter for generating CP γ photons via the VB effect. As stated above, for photons emitted in the first stage, ξ_3_ at creation is first mapped into $\xi_{1}^{\prime }$ via θ_<1, 2 >_, and then rotated to ξ_2_ via the VB effect in the second stage. This mapping is periodic with respect to θ_<1, 2 >_, and so is the induced circular polarization degree; see Figure 4c,f. For photons with $E_{\gamma }<500$ MeV, the VD purifying mechanism is relatively weak, and the circular polarization degree reaches 30% for θ_<1, 2 >_ ∈ [30°, 45°] or [135°, 150°]. For photons with $E_{\gamma }≳1$ GeV, the purifying mechanism amplifies the circular polarization degree at θ_<1, 2 >_ ≃ 30° or 150°, beyond the results corresponding to θ_<1, 2 >_ ≃ 45° or 135°. However, as shown in Figure 4c, an anomalous polarization regime (within the black circle) exhibits a circular polarization degree opposite that of its neighboring region. This anomalous CP reversal also originates from the NBW process. For θ_<1, 2 >_ > 45°, ${\bar{\xi^{\prime }}}_{3}≃\cos2\theta_{<1,2>}{\bar{\xi }}_{3}≲0$, which indicates that photons with ξ_3_ = 1 exhibit larger pair‐production rates than that with ξ_3_ = −1. Although initially *N*_γ_(ξ_3_ = 1) > *N*_γ_(ξ_3_ = −1), this inequality becomes weak with the increase in photon energy and may reverse due to pair production. For *N*_γ_(ξ_3_ = 1) < *N*_γ_(ξ_3_ = −1), ${\bar{\xi^{\prime }}}_{1}≃{\bar{\xi }}_{3}\sin2\theta_{<1,2>}\propto \left[N_{\gamma }\left(\xi_{3}=1\right)-N_{\gamma }\left(\xi_{3}=-1\right)\right]\sin2\theta_{<1,2>}<0$, i.e., ${\bar{\xi }}_{2}$ will reverse sign; see the region of $E_{\gamma }\in \left[2,3.5\right]$ GeV and θ_<1, 2 >_ ∈ [45°, 90°] in Figure 4c. The same analysis applies to the region of $E_{\gamma }\in \left[2,3.5\right]$ GeV and θ_<1, 2 >_ ∈ [90°, 135°]. Besides, since the total laser intensity is the same, the number of generated photons is relatively stable. Moreover, as mentioned previously, when θ_<1, 2 >_ ≃ 0°, the linear polarization can reach the maximum of about 80%; see Figure 4f. This is due to that the linear polarization can also be enhanced by the VD effect; see more details in ref. [68]. As the parameters are further optimized, the linear polarization degree can be even higher. We have also studied the impact of the longitudinal misalignment between the colliding point and the second subpulse focal plane. For a misalignment of ≲ 5 µm, the impact on the VB effect and photon yield is negligible (only reduce the final circular polarization by approximately 1%); see in ref. [68]. Therefore, this scheme is robust with respect to laser and electron parameters. In addition, since both the laser and electron beam parameters can affect the yields and polarization of the photon, a tradeoff should be done to fulfill the requirements of some specific applications.

## 4 Conclusion

In conclusion, we put forward a novel method for generating high‐brilliance LP and CP γ‐photon beams via the VD‐assisted VB effect, which has significant applications in material physics, nuclear physics, astrophysics, high‐energy particle physics, and new physics beyond the Standard Model, etc. Another particularly intriguing outcome of our method is the potential confirmation of the well‐known VB effect itself, which, as a cornerstone of QED and fundamental physics, was predicted more than eighty years ago but has not been directly observed in experiments yet.

## Conflict of Interest

The authors declare no conflict of interest.

## Supporting information

Supporting Information

## Data Availability

The data that support the findings of this study are available from the corresponding author upon reasonable request.
