# Supplementary material for: Generation of High‐Brilliance Polarized γ‐Rays Via Vacuum Dichroism‐Assisted Vacuum Birefringence
Source: Adv Sci (Weinh). 2025 Jul 10;12(33):e17201. doi: 10.1002/advs.202417201 (PMC12412587; doi:10.1002/advs.202417201)
Supplement: Supplementary file 1 — Supporting Information [file ADVS-12-e17201-s001.pdf]

ADVANCED SCIENCE
Open Access

Supporting Information

for *Adv. Sci.*, DOI 10.1002/advs.202417201

Generation of High-Brilliance Polarized γ-Rays Via Vacuum Dichroism-Assisted Vacuum Birefringence

Chong Lv, Feng Wan, Yousef I. Salamin, Qian Zhao, Mamutjan Ababekri, Ruirui Xu and Jian-Xing Li**

Supplemental Material

I. REFRACTIVE INDEX

Here, we quote Eq. (4.26) of Ref. [66] and include the electric field. The refractive index n for a photon with arbitrary energy ω_γ in a constant weak electromagnetic field $E, B \ll E_{cr}$ becomes (employing natural units $c = \hbar = 1$)

$$n \approx 1 - \frac{\alpha\chi_\gamma^2 m^2}{16\pi\omega_\gamma^2}\int_{-1}^{1} d\upsilon(1-\upsilon^2)\left\{\begin{matrix}\frac{1}{2}(1+\frac{1}{3}\upsilon^2)\\ 1-\frac{1}{3}\upsilon^2\end{matrix}\right\} \times\left[\pi x^{4/3}\mathrm{Gi}'(x^{2/3}) - i\frac{x^2}{\sqrt{3}}K_{2/3}\left(\frac{2}{3}x\right)\right], \quad (1)$$

where E_{cr} is the Schwinger critical field, α the fine structure constant, m the electron mass, $\chi_\gamma = e\sqrt{-(F_{\mu\nu}k^\nu)^2}/m^3 = \omega_\gamma|e\mathbf{E}_{\mathrm{red}\perp}|/m^3$ the nonlinear quantum parameter, $x = 4/[(1-\upsilon^2)\chi_\gamma]$, $\mathrm{Gi}'(x)$ the derivative of the Scorer's function, $\mathbf{K}_\nu(x)$ the νth-order modified Bessel function of the second kind, and $\mathbf{E}_{\mathrm{red}\perp} = (\mathbf{E} + \hat{k}\times\mathbf{B})_\perp$ the transverse reduced field (acceleration field for electrons). The first and second columns correspond to the eigenmodes parallel and perpendicular to $\mathbf{E}_{\mathrm{red}\perp}$, respectively. By extracting a factor of

$$\mathcal{D} = \frac{\alpha}{90\pi}\left(\frac{e|\mathbf{E}_{\mathrm{red}\perp}|}{m^2}\right)^2 \equiv \frac{\alpha}{90\pi}\frac{\chi_\gamma^2}{\omega_\gamma^2/m^2}, \quad (2)$$

Eq. (1) gives

$$\mathrm{Re}(n) = 1 - \frac{45}{4}\mathcal{D}\int_0^1 d\upsilon(1-\upsilon^2)\left\{\begin{matrix}\frac{1}{2}(1+\frac{1}{3}\upsilon^2)\\ 1-\frac{1}{3}\upsilon^2\end{matrix}\right\} \times\pi x^{4/3}\mathrm{Gi}'\left(x^{2/3}\right), \quad (3)$$

$$\mathrm{Im}(n) = \frac{45}{4}\mathcal{D}\int_0^1 d\upsilon(1-\upsilon^2)\left\{\begin{matrix}\frac{1}{2}(1+\frac{1}{3}\upsilon^2)\\ 1-\frac{1}{3}\upsilon^2\end{matrix}\right\} \times\frac{x^2}{\sqrt{3}}K_{2/3}\left(\frac{2}{3}x\right). \quad (4)$$

By defining

$$M(\chi_\gamma) = -\frac{45}{4}\int_0^1 d\upsilon(1-\upsilon^2)\left\{\begin{matrix}\frac{1}{2}(1+\frac{1}{3}\upsilon^2)\\ 1-\frac{1}{3}\upsilon^2\end{matrix}\right\}\pi x^{4/3}\mathrm{Gi}'\left(x^{2/3}\right), \quad (5)$$

one has

$$\mathrm{Re}(n) = 1 + M(\chi_\gamma)\mathcal{D} \equiv 1 + M(\chi_\gamma)\frac{\alpha}{90\pi}\frac{\chi_\gamma^2}{\omega_\gamma^2/m^2} \quad (6)$$

and $\mathcal{D} \equiv \frac{\alpha}{90\pi}\frac{\chi_\gamma^2}{\omega_\gamma^2/m^2}$.

In the limit of $\chi_\gamma \ll 1$, the real part simplifies to

$$\mathrm{Re}(n) = 1 + \mathcal{D}\left\{\begin{matrix}4_\parallel\\ 7_\perp\end{matrix}\right\}, \quad (7)$$

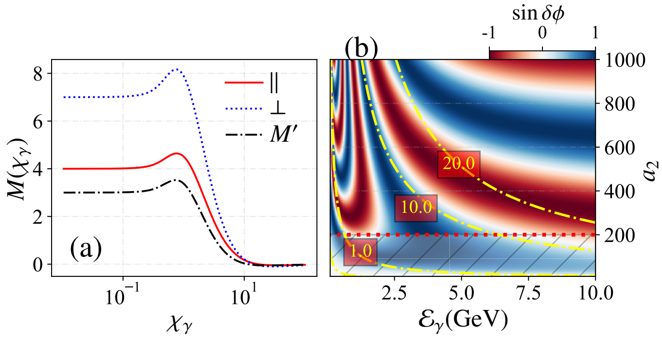

FIG. S1. (a): Two components of $M(\chi_\gamma)$ and $M' \equiv M_\perp - M_\parallel$. (b): $\sin\delta\phi$ (in a static field) vs peak field intensity a_2 (i.e., the intensity of the second subpulse in the paper) and photon energy $\mathcal{E}_\gamma$ with phase retardation $\delta\phi \equiv \frac{2\pi l}{\lambda_\gamma}\Delta n = \frac{2\pi l}{\lambda_\gamma}M'\mathcal{D}$ and photon propagation length $l = 10$ μm, where yellow contour lines indicate χ_γ, red line indicates $a_2 = 200$, and strip-band indicate the parameters used in the paper.

with $\parallel$ and $\perp$ denoting the modes parallel and perpendicular to $\hat{e}_1 \equiv \frac{\mathbf{E}_{\mathrm{red}\perp}}{|\mathbf{E}_{\mathrm{red}\perp}|}$, respectively. Figure S1(a) shows that the nonlinear terms $M(\chi_\gamma)$ in the refractive indexes $n_\perp$ and $n_\parallel$ (as well as M') are nearly constant for weak interactions with $M_{\parallel,\perp} \simeq 4$ and 7. They reach maximum values around $\chi_\gamma \simeq 1$, then quickly drop due to the anomalous dispersion effects [79]. Hence, to enhance the VB effect, χ_γ should be close to 1. Meanwhile, as $\delta\phi \propto \omega_\gamma\Delta n l \propto \omega_\gamma\mathcal{D}M'l$ (where l denotes the propagation length, and $\mathcal{D} \propto E_{\mathrm{red}\perp}^2$), $\delta\phi$ is not solely determined by χ_γ. This is evident in the relationship between $\sin\delta\phi$, laser intensity a_2 (where the field intensity is normalized by $mc\omega/e$ with $\omega = 2\pi c/\lambda$ and $\lambda = 1\mu$m), and photon energy $\mathcal{E}_\gamma$ in Fig. S1(b). As $\sin\delta\phi$ may change sign, it can lead to a low circular polarization degree. When one of the laser or electron beam parameters is fixed, the others should be carefully designed. Currently, the most intense laser can achieve a peak intensity of about $a_0 \simeq 300$, therefore, the optimal photon energy to produce the VB effect is about 2.5-4 GeV; see Fig. S1(b). This conclusion is also confirmed in Figs. 2(d) and (g) of the paper, where $\bar{\xi}_2$ reaches the maximum at photon energies around 2 GeV.

II. IMPACT OF THE LASER INTENSITY AND ELECTRON ENERGY ON CIRCULAR POLARIZATION DEGREE OF THE GENERATED γ-PHOTONS BEAM

Other key parameters studied in our proposal include the $\bar{\xi}_2$-dependence on the intensity of the first subpulse a_1 and the electron energy $\mathcal{E}_e$, as shown in Fig. S2. For a fixed total intensity of $a_0 = \sqrt{a_1^2 + a_2^2}$, the optimal a_1 is in the range of $30-70$; see Figs. S2(a) and (d). Much lower a_1 will yield too few nonlinear Compton photons in the

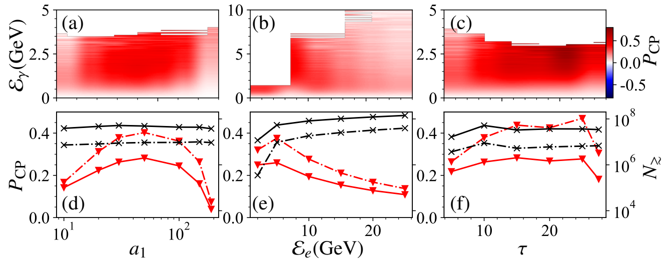

FIG. S2. Impact of the laser and electron beam parameters on the circular polarization of the generated γ-photon beam with respect to [(a) and (d)] first subpulse intensity a_1 (with the same total intensity a_0), [(b) and (e)] energy of the electron bunch $\mathcal{E}_e$ (GeV), and [(c) and (f)] pulse duration of the two subpulses (with the fixed laser energy). Here, solid and dash-dotted lines denote the results of photons with energies $\mathcal{E}_\gamma \gtrsim$ 500 MeV and 1000 MeV, respectively. All results are collected within a transverse angle of $|\theta_{x,y}| \leq 5$ mrad. Red and black lines indicate $P_{\rm CP}$ and the total number $N_{\gtrsim}$. All other parameters are the same as those in Fig. 2 of the paper with an incidence of 6.25×10^7, i.e., with a total charge of 10 pC.

first subpulse, and more photons are generated in the second subpulse. However, photons generated in the second subpulse cannot be transformed into circularly polarized (CP) ones. In contrast, a larger a_1 will induce a smaller a_2, thereby suppressing the VB effect, i.e., reducing the circular polarization degree. As the total intensity a_0 is maintained, the number of photons is stable with respect to a_1. In Figs. S2(b) and (e), $\overline{\xi}_2$ reaches its maximum around $\mathcal{E}_e \simeq 5$ GeV. As the VB effect strongly depends on χ_γ, low-energy electrons will emit low-energy photons and yield a low circular polarization degree. In the case of using high-energy electrons, such as 20 GeV, the VB effect also decreases due to the anomalous dispersion effect, and more photons decay into e^+e^- pairs. For cases of fixed total energy, the pulse duration and intensity can be varied; see results in Figs. S2(c) and (f). When the total laser energy remains constant, the total photon number yield for pulse durations in the range of $\tau \in [5, 60]T_0$ remains quite stable with small variation. As shown in Fig. S1(a), $M'(\chi)$ is constant for $\chi \lesssim 0.1$, reaches a maximum around $\chi \simeq 0.2$, and then decreases dramatically for larger χ. Consequently, for a laser with fixed energy and focal radius, the intensity decreases with increasing τ, and hence reduces χ for each photon. For high-energy photons with $\mathcal{E}_\gamma \gtrsim 500$ MeV, $\chi \gtrsim 2a_2 \frac{\mathcal{E}_\gamma}{m_ec^2}\frac{\hbar\omega_0}{m_ec^2} \gtrsim 5 \times 10^{-3} a_2$. For $a_2 \simeq 200$, $\chi \gtrsim 1$, and the VB effect continues to increase with decreasing a_2 as $\delta\phi \propto M' a_2^2 \tau$, until χ reaches 0.2 ($a_2 \simeq 40$), where $M' \simeq 3$. However, with increasing τ, the intensity of both subpulses will decrease, which will then lead to a reduction in photon yield in the first subpulse. Therefore, this significantly diminishes the VB effect. Thus, for $\tau \gtrsim 25T_0$, the circular polarization starts to decrease.

In the paper, we show that the nonlinear Breit-Wheeler (NBW, i.e., vacuum dichroism, VD) process can act as a polarization purification to enhance the VB effect. In Figs. S2(a) and (c), the peak $\overline{\xi}_2$ is in the vicinity of $a_1 \simeq 50$. Here we show that the purification mechanism also plays an important role in the case of $a_1 = 50$. For the high-energy part of the photon energy spectra, for instance, $\mathcal{E}_\gamma \gtrsim 2$ GeV, the purifying mechanism can enhance the circular polarization degree from 35% (45°) to 45% (30°), i.e., nearly a magnitude of 30%; see Fig. S3.

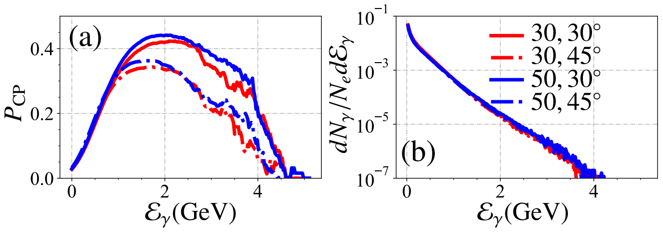

FIG. S3. (a) and (b): Energy-dependent $P_{\rm CP}$ and the corresponding energy spectra, labeled with $(a_1, \theta_{<1,2>})$. Red and blue lines indicate results from cases of $a_1 = 30$ and 50, respectively. Solid and dash-dotted lines indicate $\theta_{<1,2>} = 30°$ and 45°, respectively. All other parameters are the same as those in Fig. 2 of the paper.

III. IMPACT OF THE DISTANCE BETWEEN THE TWO SUBPULSES FOCAL POSITION

The angle distribution of all photons is presented in Fig. S4, with (a) denoting photons generated by the primary electrons, (b) and (c) denoting photons generated by the pair electrons and pair positrons, respectively. Compared with photons generated by pairs, the photons generated by primary electrons not only outnumber them by approximately 4 orders of magnitude but also exhibit a much narrower angular distribution, with a FWHM of about $\Delta\theta = 2.7$ mrad (see Fig. S4(d)). Therefore, in our work, the photons witnessed by the second subpulse mainly come from the collision between the electron beam (noted as "primaries") and the first subpulse, and the divergence angle of these photons is relatively small; see Figs. S4(a) and (d).

Besides, we have calculated and obtained the number of photons within the radius $r_0 = 3$ μm by varying the distance δl between the photons and the second subpulse; see results in Fig. S5. When $\delta l = 0$, all photons can be captured by the second subpulse, and the corresponding number of photons is about 6.83×10^8 (100.0%); see Fig. S5(a). If the collision distance is further increased, the corresponding numbers of γ photons that could be witnessed by the second subpulse are about 6.58×10^8 (96.3%) for $\delta l = 0.1$ mm, 6.02×10^8 (88.2%) for $\delta l = 1.0$ mm, and 9.56×10^7 (14.0%) for $\delta l = 10.0$ mm, respectively; see Figs. S5(b)-(d).

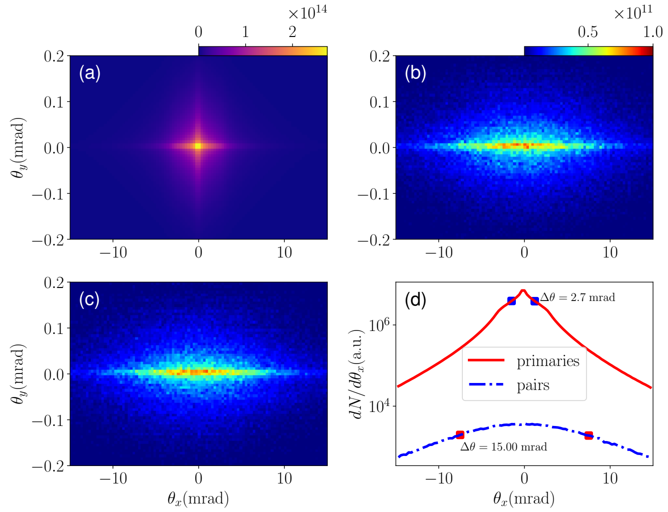

FIG. S4. Angular distribution of the generated photons $d^2N/d\theta_x d\theta_y$ (mrad^{-2}) via the scattering the first subpulse with primary electron beam [(a)] and with generated e^+e^- pairs [(b) and (c)], where $\theta_{x,y} \equiv p_{x,y}/p_z$. (d): Number distribution of generated photons with respect to θ_x.

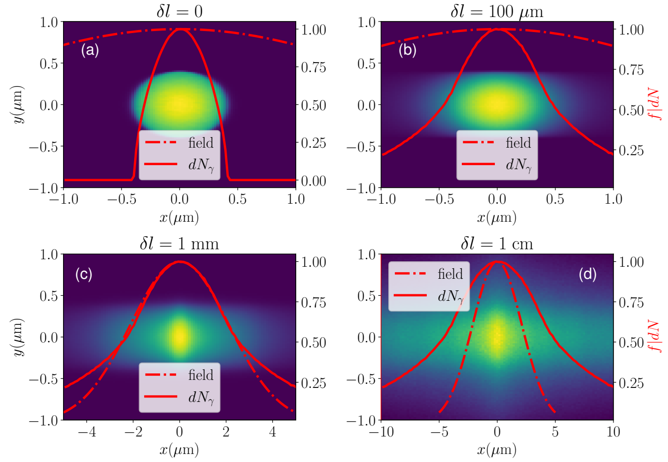

FIG. S5. The spatial number distribution of the photon generated in the first subpulse after propagating different distances (δl) (a) δl = 0, (b) δl = 0.1 mm, (c) δl = 1.0 mm, and (d) δl = 10.0 mm, respectively. The red solid line represents the normalized number distribution of photons with respect to x-axis, while the red dashed line represents the normalized distribution of laser fields f with respect to x-axis.

IV. IMPACT OF DEFLECTION OF THE PRIMARY ELECTRONS

The deflection of electrons by magnets and their impact on circular polarization are discussed below. The divergence angle $\delta\theta$ of the γ-photon beam generated in the first subpulse is on the order of $a_1/\gamma_e \simeq 3$ mrad. To ensure that these photons can scatter from the second subpulse (with focal radius $w \lesssim 3$ μm), the distance δz between two scattering points (i.e. the temporal delay between the two subpulses) should be smaller than $w/\delta\theta \simeq 1$ mm. We assume a pair of permanent magnets to deflect the primary electrons. The deflection angle and transverse position can be approximated by $\delta\theta' \simeq \frac{eB\delta z}{\gamma m_e c} \gtrsim \delta\theta$, and $\delta l \simeq \frac{\sqrt{p_0^2 - e^2B^2\delta z^2} - p_0}{eB} \gtrsim w$ (and $\delta z \lesssim w/\theta$), respectively, where B is the deflection magnetic field and p_0 the initial momentum of the primary electrons. This requires B to be on the order of 100 T and even higher (for electrons of 5 GeV), which is a great challenge for currently available magnets.

However, the simulation results indicate that the deflection does not significantly change the final circular and linear polarization degree of the generated γ-photon beam; see the comparison between the cases of with and without deflecting the primary electrons in Fig. S6.

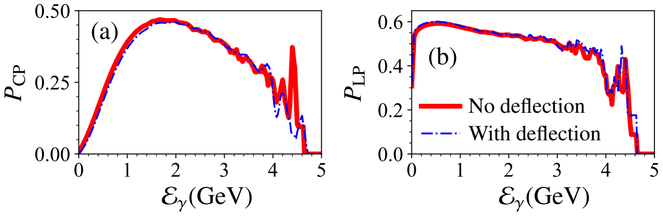

FIG. S6. Comparison of the photon polarization with and without deflection of the primary electrons. (a) and (b): circular and linear polarization, with red solid and blue dashed-dotted lines indicating cases of without and with deflection of primary electrons. All other parameters are the same as those in Fig. 2 of the paper.

V. IMPACT OF THE LONGITUDINAL MISALIGNMENT BETWEEN THE COLLIDING POINT AND THE FOCAL PLANE, AND THE FOCUSING EFFECT ON THE VB EFFECT

In the actual ultra-intense laser experiments, synchronization issues may lead to misalignment between the colliding point and the focal plane of the laser pulse. This misalignment may suppress the VB effect as indicated in Ref. [64]. When γ photons interact with the focused subpulse and the colliding point does not coincide with the focal plane, they are unable to experience the peak intensity of the laser pulse, which may influence the VB effect. We show that a misalignment of $z' \lesssim 10$ μm will reduce the final circular polarization of approximately 2%-3%; see Fig. S7. And when $z' \lesssim 5$ μm, the impact of the misalignment is negligible; see Fig. S7(b).

We have also compared the results of the focused pulse (from the paper) and the plane-wave pulse. One can note that the circular polarization and photon yields are almost identical for both cases; see Figs. S8(a) and (c). However, for cases with a much longer pulse duration of $\tau \gtrsim 40T_0$, the circular polarization and photon yields of

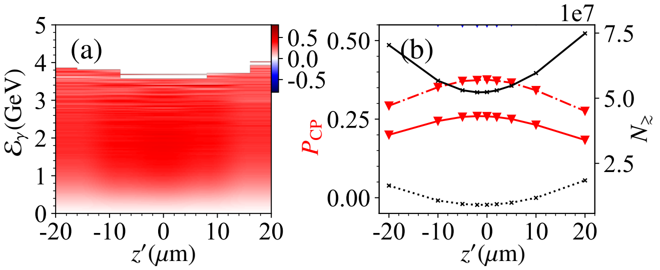

FIG. S7. Impact of the longitudinal misalignment (z') between the second subpulse and the γ photon beam on the circularly polarized γ photon yields and polarization. (a): circular polarization $\overline{\xi}_2$ vs photon energy $\mathcal{E}_\gamma$ and misalignment z'. (b): red and black lines indicate the circular polarization and photon number yields, respectively. Here, solid and dash-dotted lines denote photons with $\mathcal{E}_\gamma \gtrsim 500$ MeV, and $\mathcal{E}_\gamma \gtrsim 1$ GeV, respectively. All results are collected within an angle of $|\theta_{x,y}| \leq 5$ mrad. All other parameters are the same as those in the Fig. 2 in the paper.

the focused pulse are higher than those in the case of the plane-wave pulse; see Fig. S8(b). As mentioned in the paper, for subpulse with a duration of an overlong duration, on the one hand, it will consume more photons via the e^+e^- pair production. On the other hand, these e^+e^- pairs will create more photons (but can not be transformed into circularly polarized ones from the linearly polarized photons) in the second subpulse. For the case of a focused laser, the laser intensity will decrease due to the diffraction effect and therefore gains a larger VB effect and more photon yields.

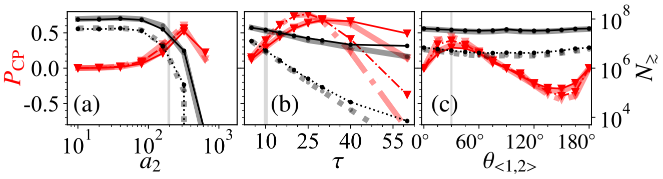

FIG. S8. Comparison of the results from focused and plane-wave pulses with respect to (a) intensity of the subpulse a_2, (b) laser pulse duration τ, and (c) relative polarization angle $\theta_{<1,2>}$. All results are collected within an angle of $|\theta_{x,y}| \leq 5$ mrad. Red and black lines indicate P_{CP}, $P_{\rm LP}$, and the total number $N_\gtrsim$. The vertical gray lines indicate parameters used in Figs. 2 and 3 of the paper. All other parameters are the same as those in Fig. 2 of the paper. Thin and thick lines denote the same quantities with thin lines are identical to Figs. 4(d)-(f) of the paper, and light-thick lines are calculated from the plane-wave pulse.

VI. SIGNAL FOR THE DETECTION OF THE VB EFFECT

As mentioned in the paper, our proposed method for generating CP γ-photon beams also serves as an alternative approach to detect the VB effect. In Fig. S9(a),

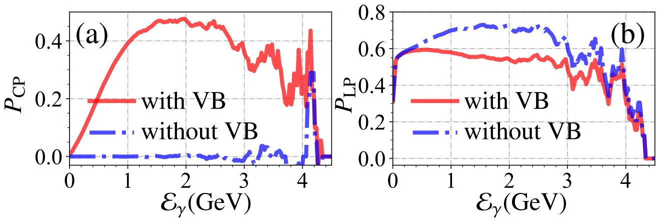

FIG. S9. Signal for the VB effect: (a) circular polarization, and (b) linear polarization of the generated γ-photon beam. Blue dash-dotted lines indicate without the VB effect. All other parameters are the same as those in Fig. 2 of the paper.

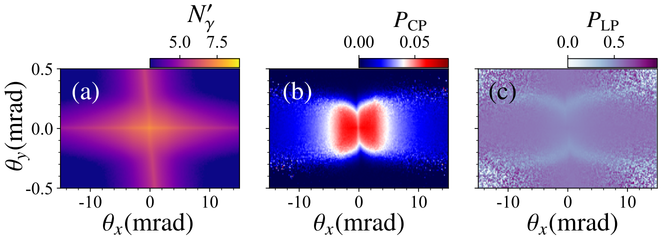

FIG. S10. (a)-(c): Angular distribution of the photon number $N'_\gamma \equiv \log_{10} d^2N_\gamma/d\theta_x d\theta_y$, circular polarization $P_{\rm CP}$, and linear polarization $P_{\rm LP}$ with respect to θ_x and θ_y. Here, all photons are collected in the full energy band.

the circular polarization can only occur in the case of including the VB effect. To detect the VB effect, the polarization-dependent Bethe-Heitler process is usually employed to measure the change in linear polarization of the γ-photon beam. As shown in Fig. S9(b), in the energy range of $\mathcal{E}_\gamma \in [1, 3]$ GeV, due to the VB effect, the decrease in linear polarization degree can reach about $\delta\overline{P_{\rm LP}} \simeq 15\%$ with a photon yield of the order of 10^6 (with 10 pC primary electrons). Due to the wide energy spectra, the VB detection accuracy of this scheme may not compete with those existing proposals with the same number of photons. However, since the photon yield per shot is 2 to 3 orders higher than that of the linear Compton source, our method can provide a detection efficiency comparable to other methods [53, 54].

VII. ANGULAR DISTRIBUTION OF THE γ PHOTONS WITH FULL ENERGY RANGE

In Figs. 2(a)-(c) of the paper, we limit the photon energies to 500-3000 MeV. Here, the angular distribution of the photon number with the full energy range is presented in Fig. S10. Without energy filtering, owing to the large population of low-energy photons, the circular polarization will decrease and the linear one will increase.

VIII. THE VD EFFECT ENHANCING THE LINEAR POLARIZATION

In Figs. 2(f) and 4(f) of the paper, we show that the VD effect can also enhance the linear polarization of the generated γ-photon beam. With current laser and electron beam parameters, the maximum linear polarization can reach about 80%; see the blue line with $\theta_{<1,2>} \simeq 0, \pi$ (i.e., the polarization of the first and second subpulse is identical) in Fig. 4(f) of the paper. The enhanced mechanism is presented in Fig. S11. One can note that for the case of $\theta_{<1,2>} = 0$, the asymmetric pair production (i.e., larger ratio of photons with $\xi_3 < 0$ are consumed compared with that of photons with $\xi_3 > 0$) is more obvious than that of $\theta_{<1,2>} = 30^\circ$ and 45° due to a much larger $\xi_3' = \xi_3 \cos 2\theta_{<1,2>} = \pm 1$ ($\xi_3' \simeq \pm 1/2$ and 0 for $\theta_{<1,2>} = 30^\circ$ and 45°, respectively) in the second subpulse; see Fig. S11(a). The final linear polarization increases from the original of about 50% to the final of about 80%; see Fig. S11(b). As the parameters are further optimized, the linear polarization degree can be even higher. However, due to $\bar{\xi}'_1 \simeq 0$, the VB effect is negligible.

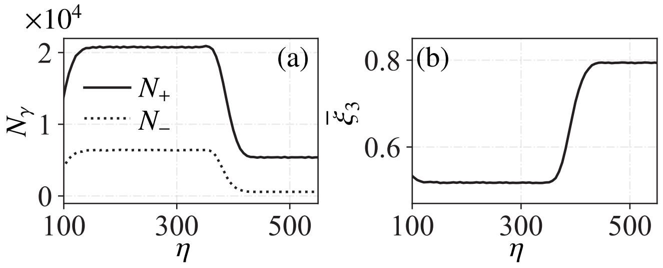

FIG. S11. (a) and (b): Evolution of photon numbers in a sampled simulation for photons with $\mathcal{E}_\gamma > 1$ GeV, where $\theta_{<1,2>} = 0$, and solid and dash-dotted lines represent photons generated with $\xi_3 > 0$ and $\xi_3 < 0$, respectively. Note that, in (a) and (b), ξ_3 is taken from the instantaneous frame. Other simulation parameters are the same as those in Fig. 2 of the paper.

IX. IMPACT OF THE LINEAR QED PROCESSES

Here, the linear processes are mainly the Compton process between the laser photons with electrons/positrons and BW processes between γ photons with the laser photons. As the photon and electrons/positrons collide with the ultraintense laser with $a \gg 1$, i.e., the primary leptons and generated leptons are all dressed in the laser field; therefore, these processes should be calculated by the one-photon absorption channel of the nonlinear processes. As shown in [18], the cross section of nonlinear Compton is given by

$$d\sigma = \frac{r_e^2}{4x} \sum_n F^{(n)} d\Gamma_n,$$

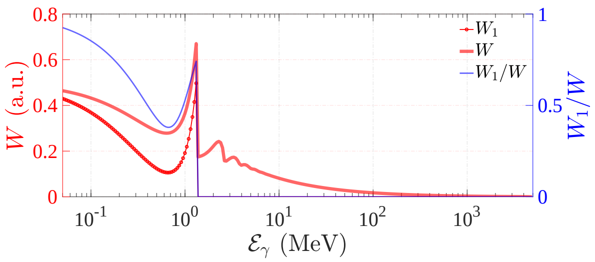

FIG. S12. Radiation probability rate of the nonlinear Compton scattering in arbitrary units. Red thick and thin (with circle as marker) lines denote the total radiation probability rate by summing over all absorption channel and the one-photon absorption channel, respectively. Blue line indicates the relative ratio of the radiation probability from first channel over the total rate.

with

$$d\Gamma = \delta(q + nk - q' - k') \frac{d^3k'}{\omega'} \frac{d^3q'}{q_0'}$$

, and n denote the number of photons absorbed. If we extract the one-photon absorption channel from the total cross section, the relative differential radiation cross section (probability) with respect to the photon energy is given in Fig. S12.

In this Fig. S12, on can see that, for emission energies smaller than the Compton edge of $4\gamma^2\hbar\omega_L \simeq 4 \times 10^6 \times 1.2eV \simeq 5$ MeV, the one-photon absorption is the dominant channel. However, for high-energy emission with $\mathcal{E}_\gamma \gg 1$ MeV, the interval of the cutoff edge of the adjacent absorption channel is harshly decreased (one can see from the vibrating of the light-red curve) and finally merged.

The nonlinear Breit-Wheeler process has the same behaviour as in the case of nonlinear Compton. The cross section in [68] is given by

$$d\sigma = \frac{r_e^2}{4x} \sum_n \bar{F}^{(n)} d\Gamma_n,$$

with $d\Gamma_n = \delta(k_1 + nk_2 - q_+ - q_-) \frac{d^3q_+}{(q_+)_0} \frac{d^3q_-}{(q_-)_0}$, and n denotes the number of absorbed photons, $\bar{F}^{(n)} = -F^{(n)}$. However, unlike the NCS, there is a threshold of the absorbed photons to generate electron positron pairs:

$$n_{th} = \frac{4(1 + a_0^2)}{x}$$

with $x = \frac{2k_1k_2}{m^2}$, for $x < x_1 \equiv 4(1 + a_0^2)$ see Eq. (10-12) in Ref. [68]. In our setup, $a_0 \gg 1$, therefore, the one γ photon with one laser photon scattering is forbidden, and the threshold $n_{th} \simeq 3.8 \times 10^4 (1.6 \times 10^6)$ for $a_0 = 30(200)$. Therefore, the single-photon channel is forbidden for the NBW process. Meanwhile, in the purely linear BW process, two conditions must be met: $\omega_1 + \omega_2 \geq 2m_ec^2$ and

$s = (k_1 + k_2)^2 > 4m_e^2$ [69]. Even though the total energy condition is satisfied, the restriction on s is the same as x in the non-linear case and therefore cannot be fulfilled. In summary, the linear BW process is not possible under the current condition.

X. PLASMA EFFECT ON THE QED PROCESS

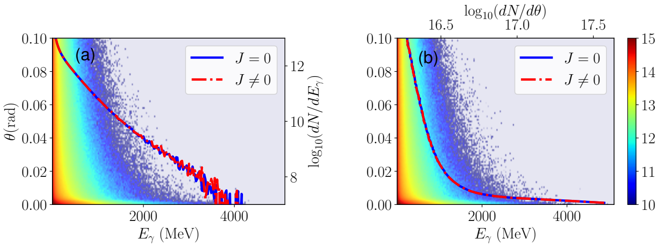

FIG. S13. (a) and (b): Number density of generated photons $\log_{10} d^2N/d\theta dE_\gamma(\mathrm{rad}^{-1}\mathrm{MeV}^{-1})$ with respect to the polar angle $\theta \equiv \arctan p_\perp/p_\parallel$ and the photon energy E_γ. (a) and (b) are calculated in the cases of turning on and off the current of all particles, i.e., considering or ignoring the self-generating fields, respectively. Blue and red lines in (a) indicating the photon density with respect to the energy for cases of ignoring and considering the self-generating field, respectively. The same line distributions are with respect to the angle in (b). All other parameters are the same as those in the Fig. 2 in the manuscript.

As the electron beam density is in the order of 10^{-3}-$10^{-2}n_c$, therefore, one should carefully examine the impact of the plasma effect, i.e., the self-generating field of the electron beam and electron-positron pairs. Here, we have done 2D simulations of the electron bunch scattering with two subpulses to compare the impact of the self-generating fields by using the PIC code EPOCH which includes the nonlinear Compton scattering and nonlinear Breit-Wheeler scattering processes. Here, all parameter settings are consistent with those used in Fig. 2 of the manuscript.
To examine the plasma effect, we have compared the results from the cases of turning the current on and off, and all other parameters are fixed; see the comparison of the photon density in Fig. S13 and the electric field components in Fig. S14, respectively. In the PIC simulation, the laser field is polarized in the y direction and propagating from the right to the left along the x direction ($y = 0$). One can see that the photon density with respect to the angle θ and energy E_γ is almost identical for cases that turn the current on or off (i.e., with/without the plasma effect); see Fig. S13. Here, Fig. S14 is taken after the electron/photon beam interacts with the second subpulse. The y components (the laser field) of both cases are almost identical; see Figs. S14(a) and (b). The differences are mainly observed in the x component, in which the laser intensity is relatively weak compared to the y component of the laser. In the region deviating from the optical axis ($y = 2.5\lambda$), the intensity of the x component of the laser field is on the order of 10^{13} V/m, exceeding the self-generated electric field by approximately two orders of magnitude. Therefore, the distribution of the field is almost identical with/without considering the plasma effect; see Fig. S14(c). In the region near the optical axis ($y \simeq 0$), the intensity of the x component of the laser field is close to zero. The intensity of the plasma-induced electric field is in the order of 10^{11} V/m, which is much lower than the intensity of the laser field ($E_y \sim 10^{14}$ V/m and $E_x \sim 10^{13}$ V/m); see Figs. S14(a)-(d). Therefore, with the current laser and electron beam parameters, the plasma effect is negligible.

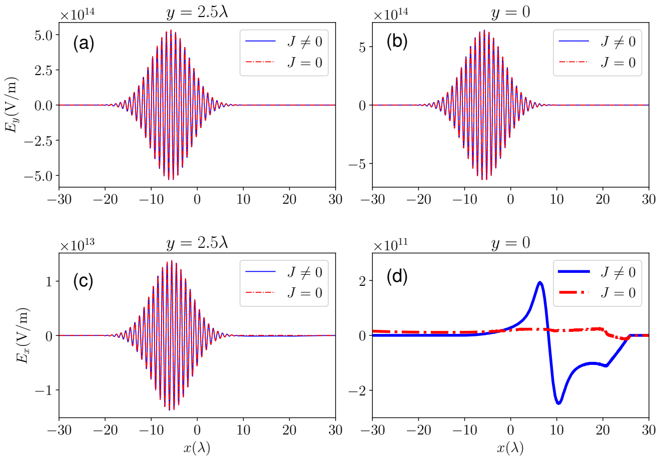

FIG. S14. 2D PIC results with all parameters are the same as those of the Fig. 2 in the manuscript. (a) and (c): E_y and E_x are taken along $y = 2.5\lambda$; (b) and (d): E_y and E_x are taken along $y = 0$. Red and blue lines denote the cases of ignoring and considering the self-generating field, respectively. In the simulation, the laser is incident from the right boundary, and the electron beam enters the simulation area from the left boundary.
